# Dosing Time Matters? Nighttime vs. Daytime Administration of Nifedipine Gastrointestinal Therapeutic System (GITS) or Amlodipine on Non-dipper Hypertension: A Randomized Controlled Trial of NARRAS

**DOI:** 10.3389/fcvm.2021.755403

**Published:** 2021-11-29

**Authors:** Jing Liu, Xiaofeng Su, Ying Nie, Zhihuan Zeng, Hongyan Chen

**Affiliations:** ^1^Department of Cardiology, Peking University People's Hospital, Beijing, China; ^2^Department of Internal Medicine, Beijing Jiaotong University Hospital, Beijing, China; ^3^Department of Cardiology, The First Affiliated Hospital of Guangdong Pharmaceutical University, Guangzhou, China; ^4^Department of Internal Medicine, North China Electric Power University Hospital, Beijing, China

**Keywords:** non-dipper hypertension, ambulatory blood pressure monitoring, pulse wave velocity, chronotherapy, randomized controlled trial

## Abstract

**Background:** Non-dipper hypertension is often characterized by a blunted decrease of nocturnal blood pressure (BP) and is associated with increased risk of target organ damage and cardiovascular (CV) events, while the optimal treatment strategy is yet to be established. This trial was designed to evaluate whether nocturnal BP reduction and arterial stiffness improvement differ from antihypertensive agents and time of administration.

**Methods:** Young and middle-aged adults (18–65 years) with non-dipper hypertension were randomly assigned to nifedipine GITS (gastrointestinal therapeutic system) 30 mg or amlodipine besylate 5 mg once daily for 8 weeks, either taken in the morning or at night. Dose was doubled at 4-week if BP is not at goal. Twenty-four hour ambulatory BP monitoring (ABPM) and arterial stiffness were evaluated before and after 8 weeks of pharmacotherapy. The primary efficacy measure was the average nighttime systolic BP reduction.

**Results:** A total of 98 non-dipper hypertensive patients (mean age 46.3 years) were randomized during Dec, 2016 and Dec, 2020, of whom 72 (73%) patients completed all ABPM and follow-up evaluations. Nighttime systolic BP significantly reduced at 8 weeks vs. baseline with nifedipine GITS or amlodipine, irrespective of dosing at nighttime (−9.9 vs −9.9 mmHg, *P* > 0.05) or daytime (−11.5 vs. −10.9 mmHg, *P* > 0.05). No difference was seen between these two agents, when combining the data of nighttime and daytime dosing together (−10.8 vs. −10.5 mmHg, respectively, *P* = 0.898). Daytime, 24-h systolic BP, diastolic BP at different time and pulse wave velocity reduced significantly and comparably, and recovery of dipping rhythm were similar among groups.

**Conclusion:** Nighttime dosing of long-acting antihypertensive preparations, nifedipine GITS or amlodipine demonstrated similar effects on nocturnal BP reduction, dipping rhythm restoration and arterial elasticity improvement in younger subjects with non-dipper hypertension. These effects were comparable with morning dosing.

## Introduction

Non-dipper hypertension is characterized by an attenuated decline of nocturnal systolic blood pressure (BP), <10% of the diurnal value, and is associated with increased risk of target organ damage and cardiovascular (CV) events, even in young and middle-aged population (1–3). No consensus and recommendations is available on how to treat non-dipper hypertension and optimal dosing time for antihypertensive medications is unclear, while it is generally accepted that a tailored BP management strategy to preserve normal 24-h circadian variations or restore dipping rhythm may have potentials on CV protection in non-dippers. On the other hand, elevated nocturnal BP is also associated with organ damage, both in dippers and non-dippers (4). This indicated that therapeutic strategies should not only address the non-dipper pattern but also focus on nocturnal BP reduction to protect hypertensive subjects. Moreover, nocturnal hypertension is more prevalent in Asians than in Europeans (5), and is associated with increased arterial stiffness in middle-aged Chinese population (6), while little is known about the impact of chronotherapy on arterial stiffening.

“Nifedipine GITS and Amlodipine besylate administrated in daytime or at nighttime on Recovery of blood pressure Rhythm and Arterial Stiffness in the young and middle-aged subjects with non-dipper hypertension” (NARRAS) was an investigator initiated randomized controlled trial (RCT), designed to explore the strategy focused on BP dipping rhythm recovery through nighttime dosing of antihypertensive agents, as well as the effect on arterial stiffness in younger non-dipper hypertensive population (7). Nifedipine gastrointestinal therapeutic system (GITS) and amlodipine, the two long-acting calcium channel blockers (CCBs) and the most frequently prescribed BP-lowering medications in China were used as comparators in the NARRAS trial.

## Methods

### Study Design

The NARRAS trial was a prospective, randomized, open-label, blinded endpoint (PROBE) study. The design and rationale of the NARRAS trial has been previously described (7). In brief, the eligible young and middle-aged adults (18–65 years) with non-dipper hypertension were assigned a unique randomization number from a pre-generated randomization number table, the drug as well as dosing time corresponding to the randomization number were allocated. Participants were randomly assigned to receive nifedipine GITS (N) 30 mg and amlodipine besylate (A) 5 mg once daily, either in the morning (referred to as N-M group and A-M group) or at night (N-N group and A-N group). The patients were instructed to make no changes to daily activities and diets and advised to take antihypertensive medications at the specified time (7 a.m. in the morning, or 10 p.m. at night) during the 8 weeks followed-up periods. The dosage of the antihypertensive drugs was doubled if office BP not at goal (<140/90 mmHg) at 4-week follow-ups. Ambulatory BP monitoring (ABPM) and biochemical assay were performed at baseline and end of the 8 week. Brachial-ankle pulse wave velocity (ba PWV), defined as the velocity of the propagation for the pulse wave from brachial to ankle artery, was also measured.

The NARRAS trial included antihypertensive treatment naïve patients or those previously treated with antihypertensive agents but discontinued for at least 2 weeks. The office systolic BP should be ≥140 mm Hg while <180 mm Hg, and/or diastolic BP ≥90 mm Hg while <110 mm Hg. The criteria of non-dipper hypertension were mean nighttime to daytime systolic BP ratio ≥ 0.9, and mean nighttime systolic BP ≥120 mm Hg in ABPM. Details of key inclusion and exclusion criteria are provided in Section A and B in the Supplementary Appendix.

Sample size calculation was referring to a reported standard deviation (SD) of nighttime systolic BP 5.6 mm Hg in Chinese non-dippers (8). One-hundred and twenty participants were expected to be enrolled in the NARRAS trial, with 80% power (two-sided) at the 5% level of significance to detect an anticipated 3 mm Hg difference in nocturnal systolic BP between treatment groups and an assumption about 10% drop-out during follow-ups, as described in the previously published paper (7). The setting of 3 mm Hg BP difference in NARRAS trial was clinically meaningful, as previous meta-analysis demonstrated that even 2 mm Hg systolic BP reduction were associated with effects on both morbidity and mortality outcomes (9). This was in line with the HARMONY trial, a crossover study designed to detect a 3 mm Hg difference in systolic BP between morning and evening dosing of antihypertensive medications (10).

The NARRAS trial was conducted in accordance with the principles of the Declaration of Helsinki. The protocol was approved by the Central Ethics Committee of Peking University People's Hospital (2016PHB013-02). Written informed consent was obtained from all participants.

The NARRAS trial was registered with clinicaltrials.gov, trial identifier NCT 02940548.

### Study Outcome

The primary efficacy measure was the average nighttime systolic BP reduction after 8 weeks administration of nifedipine GITS or amlodipine, in the morning or at night. The secondary outcome measures included proportion of recovery of dipping rhythm of BP at 8 weeks, and nighttime systolic BP reduction and proportion of recovery of dipping rhythm when comparing morning or evening dosing of antihypertensive medications. Change of ba PWV in 8 weeks was also observed.

### Statistical Analysis

Continuous variables were expressed as means with standard deviations (SDs) and categorical variables were expressed as frequencies and percentages. The primary analysis was performed using an intention-to-treat (ITT) approach among patients who had received the assigned therapy at least once after randomization. Per-protocol (PP) analysis was also conducted to testify the consistency toward ITT analysis. Missing and incomplete data will be substituted with the last observed value.

The primary efficacy measure, comparison of nighttime systolic BP reduction at the 8 weeks with morning or evening dosing of nifedipine GITS and amlodipine, was analyzed using a one-way analysis of variance (ANOVA). If there was a statistically significant difference between the means of independent groups, a *post hoc* LSD (Least Significant Difference) test would be performed. Proportion of dipper rhythm recovery at the 8 weeks with these two medications and different dosing time were analyzed using Chi-squared test or Fisher's exact test. Ambulatory BP reduction and change of PWV after treatment within each group were analyzed using paired Student's *t*-tests. Between groups comparison was performed using unpaired Student's *t*-test.

Data were analyzed using IBM SPSS Statistics version 24.0 (IBM Corp., Armonk, NY). A two-sided test was used; *P* < 0.05 were considered statistically significant.

## Results

The patient recruitment was slower than expected and follow-ups were heavily influenced with the pandemic of 2019 corona virus infectious disease (COVID-19). The trial was discontinued prematurely with 82% of the estimated sample size.

**Figure 1:**
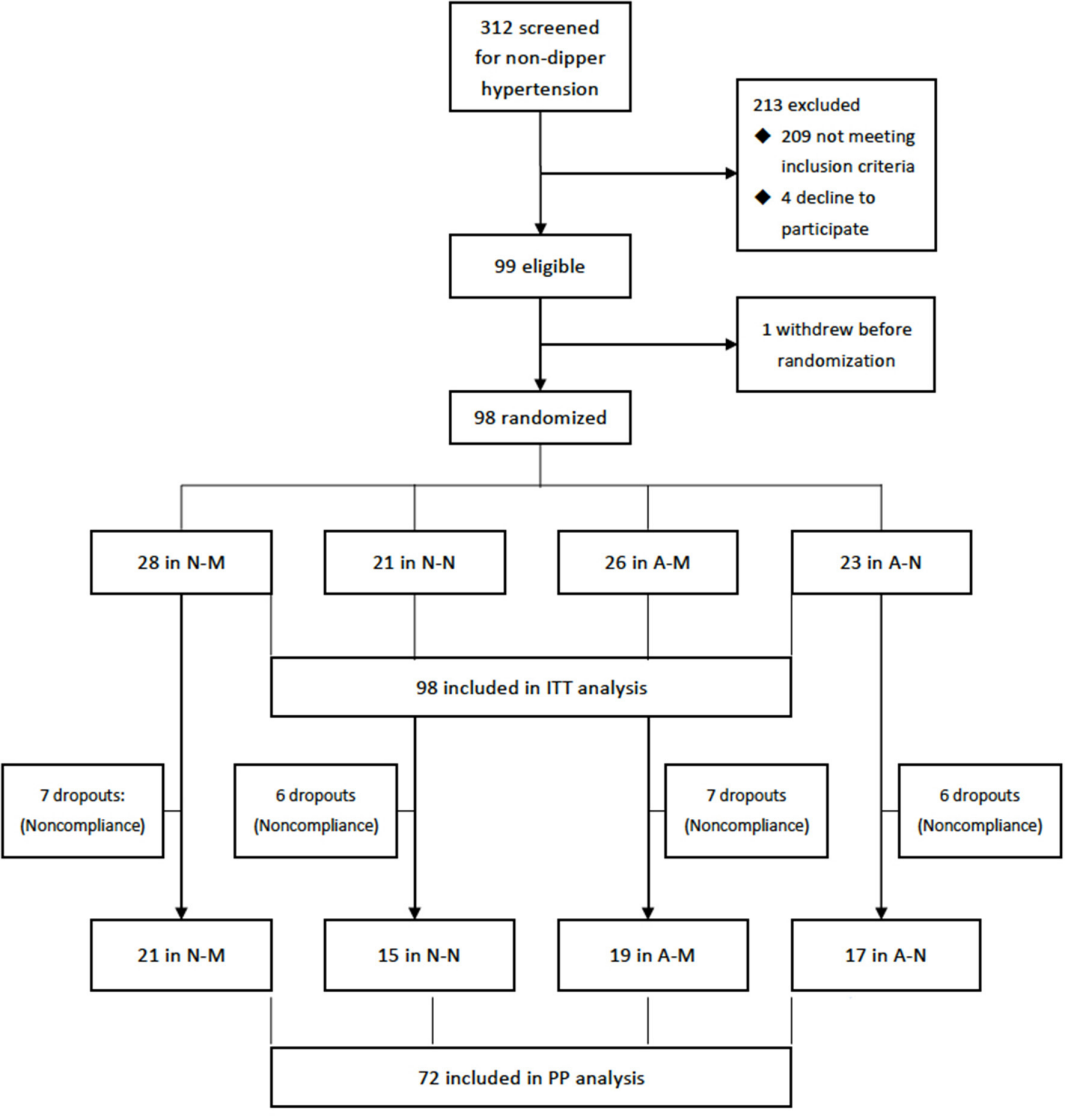
Flowchart of the trail. A-M, amlodipine in the morning; A-N, amlodipine at night; N-M, nifedipine GITS in the morning; N-M, nifedipine GITS at night; GITS, gastrointestinal therapeutic system; ITT, intention-to-treat; PP, per-protocol.

Totally, 312 hypertensive patients were screened for non-dipper hypertension and 99 eligible participants who gave informed consents were enrolled the trial and one withdrew before randomization. Ninety-eight participants (mean age, 46.3 years, 52% male) were randomized during Dec, 2016 and Dec, 2020 and included in the ITT analysis. 95 (97%) were antihypertensive treatment naïve, three patients were previously treated but discontinued by themselves 2 weeks to 2 months before inclusion. One participant provided history with rheumatoid arthritis and one with hypothyroidism, both were controlled or stable before inclusion. The others did not give information on previous history, including diabetes, coronary heart diseases, etc. Among them, 72 (74%) completed the follow-up as schedule and were included in the PP analysis (Figure 1).

Baseline characteristics were comparable among the four groups, both in the ITT analysis (Table 1) and in the PP analysis (Table 2 in Section C of the Supplementary Appendix). Detailed descriptions of the characteristics of 98 patients can be found in the Supplementary Material. Doses of medications were doubled in two participants in N-M group and one in A-M group, as office BPs were not at goal at the 4-week of follow-up.

**Table 1:** Baseline demographic and clinical characteristics of the participants.

	**N-M (*n* = 28)**	**N-N (*n* = 21)**	**A-M (*n* = 26)**	**A-N (*n* = 23)**	***P*–value**
Age (years)	46.5 ± 13.8	46.1 ± 11.7	44.5 ± 10.8	47.8 ± 9.8	0.791
Men (%)	15 (53.6)	13 (61.9)	14 (53.8)	9 (39.1)	0.491
Height (cm)	167.2 ± 6.9	167.8 ± 8.0	167.7 ± 8.8	165.7 ± 7.1	0.760
Weight (kg)	73.7 ± 12.9	74.9 ± 17.2	74.2 ± 12.4	67.3 ± 9.7	0.182
BMI (kg/m^2^)	26.3 ± 3.9	26.4 ± 4.9	26.3 ± 3.5	24.4 ± 2.2	0.211
office SBP (mmHg)	148.0 ± 8.0	148.1 ± 10.7	146.4 ± 6.8	145.7 ± 11.4	0.810
office DBP (mmHg)	95.8 ± 7.9	97.9 ± 6.1	95.3 ± 5.2	94.6 ± 6.1	0.412
Pulse rate (bpm)	75.2 ± 5.9	77.1 ± 9.8	75.6 ± 6.5	75.5 ± 7.7	0.856
24 h SBP (mmHg)	135.1 ± 7.7	139.0 ± 9.7	134.4 ± 7.3	135.8 ± 8.1	0.253
24 h DBP (mmHg)	87.2 ± 7.0	88.7 ± 7.2	87.2 ± 7.8	87.8 ± 7.7	0.888
Daytime SBP (mmHg)	136.3 ± 7.99	140.4 ± 10.6	136.8 ± 8.1	138.3 ± 8.3	0.391
Daytime DBP (mmHg)	88.7 ± 7.4	89.8 ± 7.8	89.6 ± 7.7	88.3 ± 7.0	0.894
Nighttime SBP (mmHg)	131.7 ± 8.7	134.8 ± 9.4	129.7 ± 7.5	130.3 ± 6.3	0.161
Nighttime DBP (mmHg)	82.8 ± 7.0	85.1 ± 7.2	82.8 ± 7.9	83.3 ± 8.7	0.736
PWV-Right (cm/s)	1605.7 ± 341.4	1535.6 ± 150.5	1487.6 ± 266.5	1470.5 ± 166.4	0.243
PWV-Left (cm/s)	1610.0 ± 367.3	1521.8 ± 176.1	1496.3 ± 297.1	1477.3 ± 184.9	0.346
Cr (umol/L)	63.3 ± 21.8	69.5 ± 14.5	73.0 ± 13.1	66.6 ± 13.9	0.198
UA (umol/L)	384.2 ± 109.6	398.1 ± 118.4	339.9 ± 70.2	314.0 ± 86.0	0.623
TCho (mmol/L)	5.1 ± 1.0	4.8 ± 1.0	4.8 ± 0.8	4.9 ± 1.0	0.692
HDL-C (mmol/L)	1.3 ± 0.2	1.3 ± 0.4	1.3 ± 0.3	1.3 ± 0.3	0.852
LDL-C (mmol/L)	3.2 ± 0.9	2.8 ± 0.9	3.0 ± 0.6	3.0 ± 0.8	0.299
TG (mmol/L)	1.7 ± 1.3	2.0 ± 1.6	1.6 ± 0.8	1.8 ± 1.2	0.746
Glu (mmol/L)	5.4 ± 0.7	5.8 ± 1.1	5.4 ± 0.9	5.3 ± 0.7	0.058
Cl (mmol/L)	104.0 ± 2.5	104.3 ± 3.1	104.7 ± 1.8	103.9 ± 1.7	0.636
Na (mmol/L)	140.3 ± 2.0	140.5 ± 2.4	140.0 ± 2.0	140.1 ± 1.9	0.920
K (mmol/L)	4.2 ± 0.4	4.2 ± 0.4	4.0 ± 0.3	4.1 ± 0.4	0.332

*A-M, amlodipine in the morning; A-N, amlodipine at night; N-M, nifedipine GITS in the morning; N-N, nifedipine GITS at night*.

*BMI, body mass index; Cl, chloride; Cr, creatine; DBP, diastolic blood pressure; GITS, gastrointestinal therapeutic system; Glu, glucose; HDL-C, high density lipoprotein cholesterol; K, potassium; LDL-C, low density lipoprotein cholesterol; Na, natrium; PWV, pulse wave velocity; SBP, systolic blood pressure; TCho, total cholesterol; TG, triglyceride; UA, uric acid*.

### Nighttime BP Reduction

The primary efficacy measure of nighttime systolic BP reduction from baseline were significant in each group (*P* < 0.01, respectively) and comparable in patients taken nifedipine GITS and amlodipine, irrespective of dosing in the morning or in the evening (P=0.969). That is, BP decline from 131.7 to 120.2 mmHg by the mean change of 11.5 mmHg in the nifedipine GITS morning dosing group (N-M), from 134.8 to 124.9 mmHg by mean change of 9.9 mmHg in the nifedipine GITS nighttime dosing group (N-N), from 129.7 to 118.8 mmHg by mean change of 10.9 mmHg in the amlodipine morning dosing group (A-M) and from 130.3 to 120.4 mmHg by mean change of 9.9 mmHg in the amlodipine nighttime dosing group (A-N), *P* < 0.01, respectively (Table 2). Daytime and 24-h average systolic BPs, as well as diastolic BPs decreased significantly in comparison to the baseline BPs (Table 2). No differences were found in inter-groups comparison dosing of nifedipine GITS or amlodipine (Table 3 in the Section C of Supplementary Appendix).

**Table 2:** Ambulatory BP reduction.

**BP** **(mmHg)**	**N-M (*****n*** **= 28)**			**N-N (*****n*** **= 21)**			**A-M (*****n*** **= 26)**			**A-N (*****n*** **= 23)**			**F**	***P*-value**
	**Week 0**	**Week 8**	**Changes**	**Week 0**	**Week 8**	**Changes**	**Week 0**	**Week 8**	**Changes**	**Week 0**	**Week 8**	**Changes**		
24-h systolic	135.1 ± 7.7	125.3 ± 12.9	−9.8 ± 11.9^***^	139.0 ± 9.7	131.3 ± 14.8	−7.7 ± 12.5^*^	134.4 ± 7.3	124.5 ± 7.8	−9.9 ± 10.3^***^	135.8 ± 8.1	126.9 ± 13.8	−8.9 ± 12.5^**^	0.182	0.908
24-h diastolic	87.2 ± 7.0	81.6 ± 8.0	−5.6 ± 7.4^***^	88.7 ± 7.2	85.0 ± 8.3	−3.7 ± 6.6^*^	87.2 ± 7.8	80.6 ± 6.3	−6.6 ± 7.7^***^	87.8 ± 7.7	82.6 ± 8.8	−5.2 ± 7.2^**^	0.617	0.606
Daytime systolic	136.3 ± 8.0	127.6 ± 12.7	−8.7 ± 11.5^***^	140.4 ± 10.6	134.5 ± 15.2	−5.9 ± 12.8^*^	136.8 ± 8.1	126.9 ± 8.0	−9.9 ± 10.2^***^	138.3 ± 8.3	129.9 ± 13.6	−8.4 ± 12.1^**^	0.483	0.695
Daytime diastolic	88.7 ± 7.4	83.5 ± 7.9	−5.2 ± 7.4^***^	89.8 ± 7.8	87.6 ± 9.2	−2.2 ± 7.6	89.6 ± 7.7	82.7 ± 6.6	−6.9 ± 7.4^***^	88.3 ± 7.0	84.9 ± 9.3	−3.4 ± 7.4^*^	1.749	0.163
Nighttime systolic	131.7 ± 8.7	120.2 ± 14.8	−11.5 ± 13.3^***^	134.8 ± 9.4	124.9 ± 15.4	−9.9 ± 14.7^**^	129.7 ± 7.5	118.8 ± 9.0	−10.9 ± 12.7^***^	130.3 ± 6.3	120.4 ± 14.9	−9.9 ± 13.9^**^	0.083	0.969
Nighttime diastolic	82.8 ± 7.0	77.1 ± 9.4	−5.7 ± 8.3^***^	85.1 ± 7.2	79.2 ± 8.3	−5.9 ± 8.4^**^	82.8 ± 7.9	75.9 ± 6.5	−6.9 ± 9.1^***^	83.3 ± 8.7	77.6 ± 8.8	−5.7 ± 8.0^**^	0.113	0.952

*A-M, amlodipine in the morning; A-N, amlodipine at night; N-M, nifedipine GITS in the morning; N-N, nifedipine GITS at night*.

*BP, blood pressure; GITS, gastrointestinal therapeutic system*.

*** *P < = 0.001*,

** *P < 0.01*,

* *P < 0.05*.

When comparing the combined the data of those patients taking nifedipine GITS (N-M + N-N) with that of amlodipine (A-M + A-N), no difference was found between these two antihypertensive agents (nighttime BP reduction 10.8 vs. 10.5 mmHg, *P* = 0.898) (Table 3).

**Table 3:** Comparison of ambulatory BP reduction between nifedipine GITS and amlodipine.

**BP (mmHg)**	**Nifedipine GITS (*****n*** **= 49)**			**Amlodipine (*****n*** **= 49)**			***P*-value**
	**Week 0**	**Week 8**	**Changes**	**Week 0**	**Week 8**	**Changes**	
24-h systolic	136.8 ± 8.8	128.0 ± 14.0	−8.8 ± 12.1^***^	135.0 ± 7.6	125.6 ± 10.9	−9.4 ± 11.2^***^	0.807
24-h diastolic	87.8 ± 7.1	83.1 ± 8.2	−4.7 ± 7.1^***^	87.4 ± 7.7	81.5 ± 7.5	−5.9 ± 7.4^***^	0.424
Daytime systolic	138.1 ± 9.4	130.6 ± 14.2	−7.5 ± 12.0^***^	137.5 ± 8.2	128.3 ± 10.9	−9.2 ± 11.0^***^	0.464
Daytime diastolic	89.2 ± 7.5	85.3 ± 8.7	−3.9 ± 7.5^***^	89.0 ± 7.4	83.7 ± 7.9	−5.3 ± 7.6^***^	0.381
Nighttime systolic	133.1 ± 9.0	122.3 ± 15.1	−10.8 ± 13.8^***^	130.0 ± 7.0	119.5 ± 12.0	−10.5 ± 13.2^***^	0.898
Nighttime diastolic	83.8 ± 7.1	78.0 ± 8.9	−5.8 ± 8.2^***^	83.0 ± 8.2	76.7 ± 7.6	−6.4 ± 8.6^***^	0.735

*BP, blood pressure; GITS, gastrointestinal therapeutic system*.

*** *P < =0.001*.

When comparing the combined the data of those patients of daytime dosing (N-M + A-M) with that of nighttime dosing (N-N + A-N), no difference was found between these two treatment regimens (nighttime BP reduction 11.2 vs. 9.9 mmHg, *P* = 0.634) (Table 4).

**Table 4:** Comparison of ambulatory BP reduction between morning or evening dosing.

**BP (mmHg)**	**Morning (*****n*** **= 54)**			**Evening (*****n*** **= 44)**			**P-value**
	**Week 0**	**Week 8**	**Changes**	**Week 0**	**Week 8**	**Changes**	
24-h systolic	134.7 ± 7.4	124.9 ± 10.6	−9.8 ± 11.1^***^	137.3 ± 9.0	129.1 ± 14.3	−8.2 ± 12.3^***^	0.512
24-h diastolic	87.2 ± 7.3	81.1 ± 7.2	−6.1 ± 7.5^***^	88.2 ± 7.4	83.8 ± 8.5	−4.4 ± 6.9^***^	0.283
Daytime systolic	136.6 ± 8.0	127.3 ± 10.6	−9.3 ± 10.8^***^	139.3 ± 9.5	132.2 ± 14.4	−7.1 ± 12.4^***^	0.367
Daytime diastolic	89.1 ± 7.5	83.1 ± 7.3	−6.0 ± 7.4^***^	89.1 ± 7.4	86.2 ± 9.2	−2.9 ± 7.4^*^	0.039
Nighttime systolic	130.7 ± 8.2	119.5 ± 12.2	−11.2 ± 12.9^***^	132.5 ± 8.2	122.6 ± 15.2	−9.9 ± 14.1^***^	0.634
Nighttime diastolic	82.8 ± 7.4	76.5 ± 8.0	−6.3 ± 8.7^***^	84.1 ± 8.0	78.4 ± 8.5	−5.8 ± 8.1^***^	0.776

*BP, blood pressure*.

*** *P < = 0.001*,

* *P < 0.05*.

### Dipping Rhythm Recovery and Changes of Daytime to Nighttime BP Ratio

Nifedipine GITS and amlodipine, dosing in the morning or in the evening, demonstrated similar effects on dipping rhythm recovery. Eight cases in N-M group, five in N-N group, five in A-M group and six in A-N group converted from non-dipping to dipping rhythm (mean nighttime to daytime systolic BP ratio <0.9, 28.6, 23.8, 19.2 and 26.1%, respectively, *P* = 0.566). In general, 13 (26.5%) and 11 (22.4%) of the non-dippers converted to dipping patterns in patients taking nifedipine GITS and amlodipine, respectively, no matter dosing in the morning or in the evening (*P* = 0.638). Accordingly, no difference was found in dipping rhythm recovery in morning vs. evening dosing of nifedipine GITS or amlodipine (24.1 vs 25.0%, *P* = 0.916).

Daytime to nighttime systolic BP ratio demonstrated an escalation after 8 weeks treatment of nifedipine GITS dosing in the morning (N-M group, from 1.04 ± 0.05 to 1.07 ± 0.07, *P* = 0.012), but not statistically significant in N-N (from 1.04 ± 0.05 to 1.08 ± 0.07, *P* = 0.072), A-M (from 1.06 ± 0.04 to 1.07 ± 0.06, *P* = 0.224) and A-N group (1.06 ± 0.04 to 1.08 ± 0.05, *P* = 0.106). No difference was found between groups' comparison (*P* > 0.05, respectively).

### Changes of PWV

Nifedipine GITS and amlodipine, dosing in the morning or in the evening, demonstrated similar trends toward ba PWV reduction. Changes of ba PWV were 87.5 cm/s in N-M group, *P* = 0.006; 10.1 cm/s in N-N group, *P* = 0.752; 82.8 cm/s in A-M group, *P* = 0.044 and 94.9 cm/s in A-N group, *P* = 0.020, respectively (Table 5). There was no difference in inter-groups comparison (Tables 4–6 in the Section C of Supplementary Appendix).

**Table 5:** Treatment effects on PWV.

**Group**	**Week 0**	**Week 8**	**Changes**	***P*-value**
N-M (*n* = 27)	1607.9 ± 352.8 cm/s	1520.4 ± 280.1 cm/s	−87.5 ± 153.2 cm/s	0.006
N-N (*n* = 20)	1528.7 ± 161.3 cm/s	1518.6 ± 161.4 cm/s	−10.1 ± 140.9 cm/s	0.752
A-M (*n* = 24)	1491.9 ± 280.8 cm/s	1409.2 ± 193.9 cm/s	−82.7 ± 190.1 cm/s	0.044
A-N (*n* = 21)	1473.9 ± 174.6 cm/s	1379.0 ± 217.3 cm/s	−94.9 ± 172.4 cm/s	0.020

*A-M, amlodipine in the morning; A-N, amlodipine at night; N-M, nifedipine GITS in the morning; N-N, nifedipine GITS at night; GITS, gastrointestinal therapeutic system; PWV, pulse wave velocity*.

Per-protocol analysis focused on nighttime BP reduction, dipping rhythm recovery and changes of PWV produced consistent results (Tables 7–9 in the Section C of Supplementary Appendix).

Despite that no difference was found in primary efficacy measure comparison in different groups using one-way ANOVA. To avoid missing valuable information, a *post hoc* test for pairwise comparison was performed using LSD for nighttime systolic BP reduction. The results were also negative (Table 10 in the Section C of Supplementary Appendix).

### Safety Assessment

The antihypertensive medications used in NARRAS trial were generally well tolerated. Ninety-eight patients who received at least one dose of the study drug were included in the safety assessment. One patient in the N-M group reported mild palpitation, one in N-N reported mild constipation and one in A-M group reported lower limb edema. No patients discontinued the treatment. No serious adverse event occurred.

## Discussion

BP exhibits circadian variability in both normotensive and hypertensive subjects. Patients with a blunted BP reduction in the evening are recognized as non-dippers. Reverse dipping or BP elevation during sleep is considered part of this spectrum as well. The association between these circadian variations and the risk of stroke and CV events has been firstly described by O'Brien et al. in 1988 (11), and subsequently demonstrated by other studies (12). Patients with no overnight decline in BP or non-dipper hypertension are also at higher risk of developing target organ damage, such as microalbuminuria and left ventricular hypertrophy (13, 14), while the optimal strategy for treatment of non-dipper/reverse dipper hypertension and the effects of restoration of nocturnal dipping status on CV prognosis, are yet to be established (15).

A pragmatic approach with circadian BP rhythm restoration potential is bedtime dosing of antihypertensive medications. Theoretically, short half-lives medications can adversely affect the nocturnal dip when dosing in the morning, while administration of long-acting agents at bedtime may be beneficial, as peak effects will take place during sleep-time hours. In a Japanese study, which included 34 non-dipper hypertensive patients, shifting of long-acting antihypertensive drugs from morning dosing to bedtime dosing markedly reduced nocturnal BP and 71% (24/34) of the non-dippers converted to dipping patterns (16). Evening dosing of diuretics tends to have impact on sleep quality and potentially increase BP, ultimately undermining or offsetting the potential benefits on nocturnal BP reduction. Beta-blockers dosing at night slow the heart rate and may cause lethargy. Dihydropyridine CCBs are relatively safe choice for nighttime dosing.

In NARRAS trial, nighttime dosing of nifedipine GITS or amlodipine besylate had the similar effects on nocturnal BP reduction and did not demonstrate a clear advantage on dipping rhythm restoration over the routine morning administration. This is in accordance with the results of the HARMONY trial (10). The HARMONY trial was a well-designed randomized crossover study, in which eligible patients were randomized to receive their usual medications in the morning or in the evening for 12 weeks and then dosing crossed over for a further 12 weeks. The study did not find advantage of nighttime administration of antihypertensive medications on 24-h ABPM or clinic BP control, while the information on dipper or non-dipper status of the participants and classes of BP-lowering medications, were not provided (10). In a large multicentre RCT, morning or evening dosing of valsartan 320 mg were demonstrated having a similar effects on 24-h or nighttime BP reduction in overall hypertensive participants as well as in non-dippers (17). While a crossover trial in African Americans with hypertensive chronic kidney disease, which randomized BP-lowering therapy to day vs. night also failed to show a benefit for conversion to dipping status in a large group of non-dippers (18).

The drugs used in NARRAS trial were in keeping with current guidelines and consensus recommendations (19–22). Long-acting CCBs, amlodipine or nifedipine GITS, both provide sustained 24-h BP control. This, in turn, might minimize any BP difference seen in nighttime or daytime, irrespective of dosing at daytime or nighttime. Amlodipine has a long half-life at 30–50 h, which confers the high plasma concentration lasting through the whole night, even if it is taken in the morning. Morning or evening dosing of amlodipine effectively reduced diurnal systolic BPs, while the circadian profile was not greatly affected (23). The nifedipine GITS formulation provides a once-daily dosing regimen with a constant release of the drug, resulting in a smooth plasma concentration/time profile. In NARRAS trial, nifedipine GITS demonstrated a similar impact on nocturnal BP reduction and dipping rhythm recovery as compared with amlodipine, regardless of dosing at daytime or nighttime.

The findings of similar nocturnal BP reduction in NARRAS trial with nighttime dosing vs. morning dosing, however, are contrary to some previous studies that reported improvements on BP reduction, particularly with nocturnal BP lowering in non-dippers (i.e., increasing the proportion of non-dippers to dippers and daytime to night-time BP ratio) when dosing at nighttime (16, 24). The difference may be explained by the use of true long-acting antihypertensive agents, nifedipine GITS or amlodipine in NARRAS, as the BP-lowering effects of these agents in non-dippers were similar, irrespective of morning or evening dosing. While in the HALT study, nighttime dosing of doxazosin, an alpha-adrenergic blocker, markedly reduced nocturnal BP in non-dippers. The result may be, at least partly, due to the effect of regression to the mean, as the most important determinants of the effect of doxazosin were the absolute daytime or nighttime BP levels (24). In addition, when doxazosin is taken at night, the peak plasma concentrations attain within about 2–3 h and maximum reduction in BP usually occurs 2–6 h after administration.

In contrast to the timing and choice of antihypertensive medications, sleep quality and awake physical activity were found to be significantly associated with nocturnal systolic BP fall (25), indicating the potentials of improvement of sleep quality and subsequent reduction in sympathetic tone on nocturnal BP reduction, but further interventional research is warranted (26).

Arterial stiffness, an index of the rigidity of arterial wall, is associated with increased CV disease risk independent of peripheral BP (27). The CAFÉ study revealed that amlodipine-based and atenolol-based BP-lowering regimens have different effects on arterial stiffness and central pulse pressure despite similar effects on brachial artery BP (28). However, these were not recurred in NARRAS trial, as nighttime dosing of long-acting CCBs, nifedipine GITS or amlodipine demonstrated a comparable improvement on arterial stiffening. These favorable impacts were similar when compared with morning administration.

Some large-scale trials focused on the impacts of chronotherapy on BP variations, as well as CV outcomes. The Hygia trial included 19,084 patients in primary care settings of Spain, followed about 6.3 years and demonstrated that bedtime administration of ≥1 prescribed BP-lowering medications, as opposed to upon waking, significantly enhanced asleep BP decline and markedly decreased the occurrence of CV events (29). However, the ambiguous randomization process, the unexplained expansion of recruitment of subjects in contrast to the original protocol, the incredible patient retention rate and a relatively small BP difference but striking 45% reduction of CV outcome apparent at the earlier stage of the trial while allowed to follow about 6 years, made the study controversial (30, 31). The ongoing Treatment in Morning vs. Evening (TIME) trial, which plans to include 10,200 patients and follow for 5 years, is anticipated to provide further evidence on nighttime dosing of antihypertensive medications and CV prognosis (32).

The HARMONY trial included white participants as majority and only two non-white patients (one black and one South Asian), the latter are known to have lower rates of nocturnal dipping in contrast to the white. The NARRAS trial, providing the evidence of different time dosing of antihypertensive medications on nocturnal BP dipping in Chinese non-dippers, are in accordance to the results of the HARMONY trial. These findings may have implications for optimizing dosing times for antihypertensive medications.

The NARRAS trial, together with the HARMONY and other trials, supports the concept that nighttime dosing of long-acting antihypertensive medications does not necessarily lowering BP more than routine morning administration. Currently, most patients should not be advised to make a shift of taking antihypertensive medications from daytime to nighttime, even in those with non-dipping hypertension.

### Strengths and Limitations

The strength of NARRAS trial is that we investigate the impacts of nifedipine GITS and amlodipine, the two most commonly prescribed and real long-acting BP-lowering preparations on nocturnal BP reduction and dipping rhythm restoration. The property of constant 24-h BP control of both agents might diminish BP variations between day and night. The findings from NARRAS trial should not be ambitiously generalized to medications other than these drugs. In settings where short-acting or even a once-daily but incomplete 24-h action antihypertensive agents are used, dosing time may more likely have impacts on nocturnal BP reduction and dipping pattern.

Several limitations have to be addressed. Firstly, like most trials with BP monitoring, ABPM was performed only once, no matter at the start nor in the end of the NARRAS trial. This may increase the possibility of miss leading in identifying dipping or non-dipping pattern, despite the fact that it will be difficult to perform repeated ABPM in the clinical settings. Home BP monitoring (HBPM) is promising in identifying the nocturnal BP pattern with property of accessibility and reproducibility, but the reliable devices and standardized assessment conditions and measurement frequency, as well as the prognosis value of nocturnal BP measured with HBPM, are yet to be established (33, 34). Secondly, the time of antihypertensive agents administration in the trial are pre-specified, which might be inconsistent with the true sleep/waking cycle of individual. We did not record the participants' sleep conditions and other behavioral factors at baseline, thus were uncertain of the impacts on diurnal BP variations. In addition, the trial was discontinued early, which might be underpowered in differentiating the effects of antihypertensive medications or dosing times on nocturnal BP reduction. Finally, the NARRAS trial has included relatively healthy Chinese non-dipper participants, which limits the generalization of the findings to other races or ethnicities.

## Conclusion

Nighttime dosing of long-acting antihypertensive preparations, nifedipine GITS or amlodipine demonstrated similar effects on nocturnal BP reduction, dipping rhythm restoration and arterial elasticity improvement in younger subjects with non-dipper hypertension. These effects were comparable with morning dosing.

## Data Availability Statement

The original contributions presented in the study are included in the article/Supplementary Material, further inquiries can be directed to the corresponding author/s.

## Ethics Statement

The studies involving human participants were reviewed and approved by Central Ethics Committee of Peking University People's Hospital (2016PHB013-02). The patients/participants provided their written informed consent to participate in this study.

## Author Contributions

JL conceived, designed the trial, and wrote the first draft of the article. JL and XS had full access to all of the data in the trial and take responsibility for the integrity and the accuracy of the data analysis. JL, YN, ZZ, and HC contributed to the data collection. All authors contributed to the article and approved the submitted version.

## Funding

NARRAS trial is an Investigators Initiated Research (IIR), funded by Bayer HealthCare Co., Ltd: (No. 18619).

## Conflict of Interest

The authors declare that the research was conducted in the absence of any commercial or financial relationships that could be construed as a potential conflict of interest.

## Publisher's Note

All claims expressed in this article are solely those of the authors and do not necessarily represent those of their affiliated organizations, or those of the publisher, the editors and the reviewers. Any product that may be evaluated in this article, or claim that may be made by its manufacturer, is not guaranteed or endorsed by the publisher.
