# Supplementary material for: Dosing Time Matters? Nighttime vs. Daytime Administration of Nifedipine Gastrointestinal Therapeutic System (GITS) or Amlodipine on Non-dipper Hypertension: A Randomized Controlled Trial of NARRAS
Source: Front Cardiovasc Med. 2021 Nov 29;8:755403. doi: 10.3389/fcvm.2021.755403 (PMC8666540; doi:10.3389/fcvm.2021.755403)
Supplement: Supplementary file 1 [file Data_Sheet_1.pdf]

Supplementary Material

Dosing time matters? Nighttime versus daytime administration of nifedipine gastrointestinal therapeutic system (GITS) or amlodipine on non-dipper hypertension： a randomized controlled trial of NARRAS

Jing Liu, Xiaofeng Su, Ying Nie, Zhihuan Zeng, Hongyan Chen; on behalf of the NARRAS investigators

Supplementary Appendix

Section A

Inclusion criteria

(1) Men or women, aged 18-65 years;

(2) Antihypertensive treatment naive or previously treated but discontinued at least 2 weeks;

(3) Office systolic blood pressure (BP) ⩾140 mm Hg while <180 mm Hg, and/ or diastolic BP ⩾90 mm Hg while <110 mm Hg;

(4) Non-dipper hypertension: mean nighttime/ daytime systolic BP ⩾ 0.9, and mean nighttime systolic BP ⩾120 mm Hg measured with ambulatory BP monitoring (ABPM)

Section B

Exclusion criteria

(1) Known allergy to any component of nifedipine and amlodipine;

(2) Office systolic BP ⩾ 180 mm Hg and/ or diastolic BP ⩾ 110 mm Hg in the screening period;

(3) Patient who is taking calcium channel blockers (CCBs), monotherapy or combination with other BP-lowering agents；

(4) Evidences of secondary hypertension;

(5) History of cerebrovascular events, cardiac failure, serious coronary artery disease within 12 months;

(6) Type 1 diabetes mellitus (DM);

(7) Severe liver diseases or renal insufficiency;

(8) Women who are pregnant or lactating, planning for pregnancy or of childbearing potential;

(9) Night shift workers;

(10) Other reasons unsuitable to participate in the study

Section C

Table 1. **Baseline demographic and clinical characteristics of the participants (Intention-to-treat)**

	N-M (n=28)	N-N(n=21)	A-M (n=26)	A-N (n=23)	*p*
**Age (years)**	46.5±13.8	46.1±11.7	44.5±10.8	47.8±9.8	0.791
**Men (%)**	15 (53.6)	13 (61.9)	14 (53.8)	9 (39.1)	0.491
**Height (cm)**	167.2±6.9	167.8±8.0	167.7±8.8	165.7±7.1	0.760
**Weight (kg)**	73.7±12.9	74.9±17.2	74.2±12.4	67.3±9.7	0.182
**BMI (kg/m^2)**	26.3±3.9	26.4±4.9	26.3±3.5	24.4±2.2	0.211
**office SBP (mmHg)**	148.0±8.0	148.1±10.7	146.4±6.8	145.7±11.4	0.810
**office DBP (mmHg)**	95.8±7.9	97.9±6.1	95.3±5.2	94.6±6.1	0.412
**Pulse rate (bpm)**	75.2±5.9	77.1±9.8	75.6±6.5	75.5±7.7	0.856
**24h SBP (mmHg)**	135.1±7.7	139.0±9.7	134.4±7.3	135.8±8.1	0.253
**24h DBP (mmHg)**	87.2±7.0	88.67±7.2	87.2±7.8	87.8±7.7	0.888
**Daytime SBP (mmHg)**	136.3±7.99	140.38±10.6	136.8±8.1	138.3±8.3	0.391
**Daytime DBP (mmHg)**	88.7±7.4	89.8±7.8	89.6±7.7	88.3±7.0	0.894
**Nighttime SBP (mmHg)**	131.7±8.7	134.8±9.4	129.7±7.5	130.3±6.3	0.161
**Nighttime DBP (mmHg)**	82.8±7.0	85.1±7.2	82.8±7.9	83.3±8.7	0.736
**PWV-Right (cm/s)**	1605.7±341.4	1535.6±150.5	1487.6±266.5	1470.5±166.4	0.243
**PWV-Left (cm/s)**	1610.0±367.3	1521.8±176.1	1496.3±297.1	1477.3±184.9	0.346
**ALT (U/L)**	34.8±31.6	25.8±15.0	26.3±13.2	21.9±10.6	0.140
**AST (U/L)**	25.3±10.2	21.8±5.2	23.1±10.1	20.6±4.7	0.217
**TBIL (umol/L)**	15.85±7.01	14.73±5.69	14.84±7.65	13.75±5.71	0.761
**CK (mmol/L)**	77.5±15.0	81.9±35.1	84.1±26.9	83.0±47.8	0.916
**BUN (mmol/L)**	5.1±1.8	4.5±0.9	5.0±1.1	4.5±1.1	0.298
**Cr (umol/L)**	63.3±21.8	69.5±14.5	73.0±13.1	66.6±13.9	0.198
**UA (umol/L)**	384.2±109.6	398.1±118.4	339.9±70.2	314±86.0	0.623
**TCho (mmol/L)**	5.1±1.0	4.8±1.0	4.8±0.8	4.9±1.0	0.692
**HDL-C (mmol/L)**	1.3±0.2	1.3±0.4	1.3±0.3	1.3±0.3	0.852
**LDL-C (mmol/L)**	3.2±0.9	2.8±0.9	3.0±0.6	3.0±0.8	0.299
**TG (mmol/L)**	1.7±1.3	2.0±1.6	1.6±0.8	1.8±1.2	0.746
**TP (g/L)**	76.0±3.7	74.6±4.0	75.5±3.9	76.0±10.0	0.446
**Alb (g/L)**	45.4±3.0	45.6±3.3	45.5±3.0	45.1±4.0	0.895

**Glu (mmol/L)**	5.4±0.7	5.8±1.1	5.4±0.9	5.3±0.7	0.058
**Cl (mmol/L)**	104.0±2.5	104.3±3.1	104.7±1.8	103.9±1.7	0.636
**Na (mmol/L)**	140.3±2.0	140.5±2.4	140.0±2.0	140.1±1.9	0.920
**K (mmol/L)**	4.2±0.4	4.2±0.4	4.0±0.3	4.1±0.4	0.332

A-M, amlodipine in the morning; A-N, amlodipine at night; N-M, nifedipine GITS in the morning; N-N, nifedipine GITS at night

ALB, albuminuria; ALT, alanine aminotransferase; AST, aspartate transaminase; BMI, body mass index; BP, blood pressure; BUN, blood urea nitrogen; CK, creatine kinase; Cl, chloride; Cr, creatine; GITS, gastrointestinal therapeutic system; Glu, glucose; HDL-C, high density lipoprotein cholesterol; K, potassium; LDH, lactic dehydrogenase; LDL-C, low density lipoprotein cholesterol; Na, natrium; PWV, pulse wave velocity; TBil, total bilirubin; TCho, total cholesterol; TG, triglyceride; TP, total protein; UA, uric acid

Table 2. **Baseline demographic and clinical characteristics of the participants (Per-protocol)**

	N-M (n=21)	N-N (n=15)	A-M (n=19)	A-N (n=17)	P Value
**Age (years)**	48.9±12.3	49.0±10.4	42.7±10.6	51.1±7.2	0.093
**Men (%)**	10 (47.6)	10 (66.7)	9 (47.4)	7 (41.2)	0.513
**Height (cm)**	166.0±6.5	168.3±7.9	166.2±7.8	165.9±6.8	0.072
**Weight (kg)**	73.2±14.6	78.3±17.0	72.6±12.3	66.2±9.1	0.752
**BMI (kg/cm2)**	26.5±4.4	27.5±4.9	26.2±3.6	24.0±2.0	0.072
**Office systolic BP (mmHg)**	148.6±7.6	148.6±11.4	147.1±7.0	146.6±12.7	0.916
**Office diastolic BP (mmHg)**	96.1±8.2	97.57±6.9	95.5±5.6	94.3±6.7	0.633
**Pulse rate (bpm)**	75.3±5.6	79.8±9.5	76.8±6.6	76.4±8.3	0.393
**24h systolic BP (mmHg)**	136.2±7.8	141.0±9.9	136.3±7.1	135.9±8.7	0.266
**24h diastolic BP (mmHg)**	87.7±7.8	89.9±6.5	89.0±7.6	87.6±8.6	0.783
**Daytime systolic BP (mmHg)**	137.6±8.2	142.1±11.0	139.1±7.6	138.5±8.7	0.486
**Daytime diastolic BP (mmHg)**	89.4±8.2	90.8±7.8	91.6±7.1	87.76±7.4	0.465
**Nighttime systolic BP (mmHg)**	132.3±7.8	137.3±9.3	131.0±8.1	130.2±7.1	0.074
**Nighttime diastolic BP (mmHg)**	82.8±7.1	87.1±6.6	84.0±8.5	82.7±9.8	0.372
**PWV-Right (cm/s)**	1643.1±350.7	1551.8±161.4	1481.5±297.0	1504.2±166.2	0.241
**PWV-Left (cm/s)**	1656.4±386.7	1533.6±184.6	1494.1±329.5	1508.9±191.1	0.301
**ALT (U/L)**	38.4±35.7	26.8±11.9	26.7±14.4	21.3±10.4	0.111
**AST (U/L)**	26.7±11.0	22.2±4.7	23.2±11.8	20.7±4.9	0.220
**TBIL (umol/L)**	15.5±6.7	13.8±5.5	13.8±6.5	13.7±5.8	0.787
**CK (mmol/L)**	76.0±14.6	84.6±40.6	78.7±22.7	73.3±27.7	0.733
**BUN (mmol/L)**	5.4±1.8	4.6±0.7	5.1±1.2	4.7±1.0	0.257
**Cr (umol/L)**	63.8±19.8	69.3±11.7	71.8±13.2	67.0±14.8	0.452
**UA (umol/L)**	384.9±123.9	412.3±126.4	346.6±74.3	315.9±90.7	0.058
**TCho (mmol/L)**	5.2±1.1	4.9±0.9	4.9±0.9	4.9±1.0	0.797
**HDL-C (mmol/L)**	1.3±0.3	1.3±0.5	1.3±0.3	1.3±0.3	0.954
**LDL-C (mmol/L)**	3.3±1.0	2.8±0.9	3.0±0.6	2.9±0.8	0.275
**TG (mmol/L)**	1.5±1.0	2.3±1.8	1.7±0.8	1.7±0.9	0.270
**TP (g/L)**	75.4±4.0	74.1±4.4	75.4±4.6	72.9±5.2	0.352
**Alb (g/L)**	44.7±2.8	45.1±3.4	45.2±3.2	44.4±4.1	0.892
**Glu (mmol/L)**	5.4±0.7	6.0±1.2	5.5±1.0	5.3±0.6	0.124

**Cl (mmol/L)**	104.3±2.7	104.3±3.5	105.5±1.5	104.1±1.8	0.443
**Na (mmol/L)**	140.6±2.2	141.2±2.5	140.2±2.2	140.4±2.0	0.700
**K (mmol/L)**	4.2±0.4	4.1±0.3	4.1±0.3	4.1±0.3	0.825

A-M, amlodipine in the morning; A-N, amlodipine at night; N-M, nifedipine GITS in the morning; N-N, nifedipine GITS at night

ALB, albuminuria; ALT, alanine aminotransferase; AST, aspartate transaminase; BMI, body mass index; BP, blood pressure; BUN, blood urea nitrogen; CK, creatine kinase; Cl, chloride; Cr, creatine; GITS, gastrointestinal therapeutic system; Glu, glucose; HDL-C, high density lipoprotein cholesterol; K, potassium; LDH, lactic dehydrogenase; LDL-C, low density lipoprotein cholesterol; Na, natrium; PWV, pulse wave velocity; TBil, total bilirubin; TCho, total cholesterol; TG, triglyceride; TP, total protein; UA, uric acid

Table 3. **Inter-groups comparison of ambulatory BP reduction dosing of Nifedipine GITS or amlodipine (Intention-to-treat)**

BP (mmHg)	N-M (n=28)	N-N (n=21)	p Value	A-M (n=26)	A-N (n=23)	P value
**24-h systolic**	-9.8±11.9***	-7.7±12.5*	0.545	-9.9±10.3***	-8.9±12.5**	0.758
**24-h diastolic**	-5.6±7.4**	-3.7±6.6*	0.372	-6.6±7.7***	-5.2±7.2**	0.520
**Daytime systolic**	-8.7±11.5**	-5.9±12.8*	0.421	-9.9±10.2***	-8.4±12.1**	0.639
**Daytime diastolic**	-5.2±7.4**	-2.2±7.6	0.175	-6.9±7.4***	-3.4±7.4*	0.117
**Nighttime systolic**	-11.5±13.3***	-9.9±14.7**	0.692	-10.9±12.7***	-9.9±13.9**	0.793
**Nighttime diastolic**	-5.7±8.3**	-5.9±8.4**	0.950	-6.9±9.1**	-5.7±8.0**	0.646

A-M, amlodipine in the morning; A-N, amlodipine at night; N-M, nifedipine GITS in the morning; N-N, nifedipine GITS at night

BP, blood pressure; GITS, gastrointestinal therapeutic system

*** P ＜= 0.001, ** P ＜ 0.01, * P ＜ 0.05， as compared with baseline;

Nighttime systolic BP reduction: N-M vs A-M, P=0.868; N-N vs A-N, P=0.999

Table 4. **Changes of PWV (Intention-to-treat)**

	**N-M (n=27)**	**N-N (n=20)**	**A-M (n=24)**	**A-N (n=21)**
**Average PWV (cm/s)**	-87.5±153.2**	-10.10±140.9	-82.7±190.1*	-94.9±172.4*

GITS, gastrointestinal therapeutic system; PWV, pulse wave velocity

***P＜0.001; **P＜0.01; *P＜0.05， as compared with baseline;

N-M vs N-N, P=0.083; A-M vs A-N, P=0.825; N-M vs A-M, P=0.922; N-N vs A-N, P=0.094

Table 5. **Effects of nifedipine GITS and amlodipine on PWV (Intention-to-treat)**

	**Nifedipine GITS (n=47)**			**Amlodipine (n=45)**			**P value**
	Week0	Week8	Changes	Week0	Week8	Changes	
**Average PWV (cm/s)**	1574.2±287.5	1519.6±234.8	-54.6±151.5	1483.5±234.9	1395.1±203.3	-88.4±180.1**	0.331

GITS, gastrointestinal therapeutic system; PWV, pulse wave velocity

***P＜= 0.001; **P＜0.01; *P＜0.05

Table 6. **Effects of morning or evening dosing of antihypertensive agents on PWV (Intention-to-treat)**

	**Morning (n=51)**			**Evening (n=41)**			
	Week0	Week8	Changes	Week0	Week8	Changes	**P value**
**Average PWV (cm/s)**	1553.3±323.1	1468.1±247.5	-85.2±169.8***	1500.6±168.5	1447.1±202.4	-53.5±161.7*	0.365

PWV, pulse wave velocity

***P＜= 0.001; **P＜0.01; *P＜0.05

Table 7. **Ambulatory BP reduction (Per-protocol)**

BP (mmHg)	N-M (n=21)			N-N (n=15)			A-M (n=19)			A-N (n=17)		
	Week0	Week8	Changes	Week0	Week8	Changes	Week0	Week8	Changes	Week0	Week8	Changes
**24-h systolic**	136.2±7.8	123.7±13.9	-12.5±12.1***	141.0±9.9	130.3±16.9	-10.7±13.7**	136.3±7.1	122.7±8.0	-13.6±9.8***	135.9±8.7	124.5±14.5	-11.4±13.1**
**24-h diastolic**	87.7±7.8	80.6±8.6	-7.1±7.7***	89.9±6.5	84.8±8.5	-5.1±7.4*	89.0±7.6	80.0±6.2	-9.0±7.8***	87.6±8.6	80.9±9.1	-6.7±7.5**
**Daytime systolic**	137.6±8.2	126.4±14.0	-11.2±11.9***	142.1±11.0	133.9±17.4	-8.2±14.6*	139.1±7.6	125.5±8.1	-13.6±9.7***	138.5±8.7	127.7±14.3	-10.8±12.8**
**Daytime diastolic**	89.4±8.2	82.7±8.8	-6.7±7.7***	90.8±7.8	87.7±9.9	-3.1±8.9	91.6±7.1	82.3±6.5	-9.3±7.2***	87.8±7.4	83.4±9.7	-4.4±8.3*
**Nighttime systolic**	132.3±7.8	117.5±14.6	-14.8±13.3***	137.3±9.3	123.4±17.8	-13.9±15.9**	131.0±8.1	116.1±8.8	-14.9±12.7***	130.2±7.1	117.4±15.8	-12.8±14.6**
**Nighttime diastolic**	82.8±7.1	75.5±9.4	-7.3±8.8***	87.1±6.6	78.9±9.2	-8.2±8.9**	84.0±8.5	74.6±6.4	-9.4±9.5***	82.7±9.8	75.2±8.6	-7.5±8.4**

A-M, amlodipine in the morning; A-N, amlodipine at night; N-M, nifedipine GITS in the morning; N-N, nifedipine GITS at night
GITS, gastrointestinal therapeutic system
***P＜＝0.001; **P＜0.01; *P＜0.05

Table 8. **Treatment effects on dipper rhythm recovery (Per-protocol)**

	N-M (n=21)	N-N (n=15)	A-M (n=19)	A-N (n=17)
**Dippers, n (%)**	8 (38.1)	5 (33.3)	5 (26.3)	6 (35.3)

A-M, amlodipine in the morning; A-N, amlodipine at night; N-M, nifedipine GITS in the morning; N-N, nifedipine GITS at night
GITS, gastrointestinal therapeutic system
N-M vs N-N, P=1.000; A-M vs A-N, P=0.721; N-M vs A-M, P=0.511; N-N vs A-N, P=1.000

Table 9. **Treatment effects on PWV (Per-protocol)**

Group	Week0	Week8	Changes	P value
**N-M (n=21)**	1657.4±375.2 cm/s	1539.3±293.9 cm/s	-118.1±168.2 cm/s	0.005
**N-N (n=15)**	1546.4±166.1 cm/s	1533.8±167.5 cm/s	-12.6±158.5 cm/s	0.754
**A-M (n=19)**	1487.8±312.2 cm/s	1383.3±203.6 cm/s	-104.5±209.2 cm/s	0.043
**A-N (n=17)**	1506.6±177.7 cm/s	1389.4±240.44 cm/s	-117.2±185.4 cm/s	0.019

A-M, amlodipine in the morning; A-N, amlodipine at night; N-M, nifedipine GITS in the morning; N-N, nifedipine GITS at night

GITS, gastrointestinal therapeutic system; PWV, pulse wave velocity

Table 10. **Post hoc LSD test for nighttime systolic BP**

Dependent Variable	Group1	Group2	Group1-Group2	p Value
**Nighttime systolic BP**	N-M	N-N	-1.61±3.96	0.684
		A-M	-0.6±3.74	0.874
		A-N	-1.61±3.91	0.681
	N-N	N-M	1.61±3.96	0.684
		A-M	1.02±3.99	0.799
		A-N	0±4.15	0.999
	A-M	N-M	0.6±3.74	0.874
		N-N	-1.02±3.99	0.799
		A-N	-1.01±3.94	0.798
	A-N	N-M	1.61±3.91	0.681
		N-N	0±4.15	0.999
		A-M	1.01±3.94	0.798

A-M, amlodipine in the morning; A-N, amlodipine at night; N-M, nifedipine GITS in the morning; N-N, nifedipine GITS at night

BP, blood pressure; GITS, gastrointestinal therapeutic system; LSD, Least Significant Difference

Section D

NARRAS trial coordinating centers and collaborators:

Peking University People's Hospital: Jing Liu, Xiaofeng Su, Pineng Lu, Qingchun Ma

Beijing Jiaotong University Hospital: Ying Nie, Lingwei Kong, Ronghui Xia, Wenfeng Qiu, Yifei Wang, Weina Sun, Yuntao Lu, Yao Fu, Hongjun Liu

The First Affiliated Hospital of Guangdong Pharmaceutical University: Zhihuan Zeng, Tudi Li, Yusi Yao,

North China Electric Power University Hospital: Hongyan Chen, Xiuxiu Yin
